# Membrane‐destabilizing ionizable lipid empowered imaging‐guided siRNA delivery and cancer treatment

**DOI:** 10.1002/EXP.20210008

**Published:** 2021-09-01

**Authors:** Shuai Guo, Kun Li, Bo Hu, Chunhui Li, Mengjie Zhang, Abid Hussain, Xiaoxia Wang, Qiang Cheng, Feng Yang, Kun Ge, Jinchao Zhang, Jin Chang, Xing‐Jie Liang, Yuhua Weng, Yuanyu Huang

**Affiliations:** ^1^ School of Life Science, Advanced Research Institute of Multidisciplinary Science, Key Laboratory of Molecular Medicine and Biotherapy, Institute of Engineering Medicine Beijing Institute of Technology Beijing P. R. China; ^2^ Institute of Molecular Medicine, College of Future Technology Peking University Beijing P. R. China; ^3^ Department of Biochemistry Simmons Comprehensive Cancer Center The University of Texas Southwestern Medical Center Dallas Texas USA; ^4^ Howard Hughes Medical Institute, Department of Medicine, School of Medicine University of California, San Diego La Jolla California USA; ^5^ Key Laboratory of Analytical Science and Technology of Hebei Province, College of Chemistry and Environmental Science, Key Laboratory of Medicinal Chemistry and Molecular Diagnosis of the Ministry of Education Hebei University Baoding P. R. China; ^6^ School of Life Sciences, Tianjin Engineering Center of Micro Nano Biomaterials and Detection Treatment Technology Collaborative Innovation Center of Chemical Science and Engineering, Tianjin University Tianjin P. R. China; ^7^ Chinese Academy of Sciences (CAS) Center for Excellence in Nanoscience and CAS Key Laboratory for Biomedical Effects of Nanomaterials and Nanosafety National Center for Nanoscience and Technology Beijing P. R. China

**Keywords:** cancer treatment, hepatocellular carcinoma, lipid nanoparticle, lipid‐like material, MRI, siRNA

## Abstract

One of the imperative medical requirements for cancer treatment is how to establish an imaging‐guided nanocarrier that combines therapeutic and imaging agents into one system. siRNA therapeutics have shown promising prospects in controlling life‐threatening diseases. However, it is still challenging to develop siRNA formulations with excellent cellular entry capability, efficient endosomal escape, and simultaneous visualization. Herein, we fabricated multifunctional ionizable lipid nanoparticles (iLNPs) for targeted delivery of siRNA and MRI contrast agent. The iLNPs comprises DSPC, cholesterol, PEGylated lipid, contrast agent DTPA‐BSA (Gd), and ionizable lipid termed iBL0104. siRNA‐loaded iLNPs (iLNPs/siRNA) could be decorated with a tumor targeting cyclic peptide (c(GRGDSPKC)) (termed GARP), or without targeting modification (termed GAP). Data revealed that GARP/siRNA iLNPs exhibited significantly higher cellular entry efficiency than GAP/siRNA iLNPs. GARP/siRNA iLNPs rapidly and effectively escaped from endosome and lysosome after internalization. Compared with GAP/siPLK1, GARP/siPLK1 exhibited better tumor inhibition efficacy in both cell‐line derived xenograft and liver cancer patient derived xenograft murine models. In addition, GARP formulation displayed ideal MRI effect in tumor‐bearing mice, and was well tolerated by testing animals. Therefore, this study provides an excellent example for achieving imaging‐guided and tumor‐targeted siRNA delivery and cancer treatment, highlighting its promising potential for translational medicine application.

## Graphical abstract

Development of novel ionizable lipid and realization of real‐time payload visualization are of great importance for siRNA delivery and clinical translation. Herein, we fabricated multifunctional ionizable iLNPs based on a newly prepared lipid for targeted delivery of siRNA and MRI contrast agent, which achieved good siRNA transportation, well imaging effect and excellent therapeutic outcome in tumor‐bearing mice.

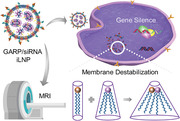

## 1 INTRODUCTION

During the past decade, the hunt for small interfering RNA (siRNA) as therapeutic modality has significantly increased.^[^ ^1^, ^2^ ^]^ Currently, there are four siRNA therapeutics that have been approved for clinical application, which include ONPATTRO^®^ (Patisiran), GIVLAARI^®^ (Givosiran), OXLUMO^®^ (Lumasiran), and LEQVIO^®^ (Inclisiran).^[^ ^3^ ^]^ Establishing an effective and clinically applicable in vivo delivery system that can mediate effective cellular entry and rapid endosomal escape is the bottle neck issue for siRNA drug development.^[^ ^4^, ^5^ ^]^ Lipid‐based nanocarriers have been extensively employed for nucleic acid delivery and clinical investigation due to their desirable properties, such as controllable preparation, high encapsulation efficiency, robust transportation efficiency, and good biocompatibility.^[^ ^4^, ^6^ ^]^ Patisiran, the first commercialized siRNA therapeutic in the world, is a lipid formulation employing Dlin‐MC3‐DMA as the determinate key lipid.^[^ ^2^, ^7^ ^]^ It is reported that when ionizable lipid stays in acidic endosome, it will be positively charged and interact with the endosomal membrane anionic lipids such as phosphatidylserine, leading to disruption of endosomal membrane, effective nucleic acid escape, and cytosolic release.^[^ ^8^, ^9^ ^]^

In addition, visualization of pathogenic sites, such as tumor tissues, is also critical for clinical treatment, which facilitates early diagnosis, drug tracking, and observation of disease progression and metastasis. Magnetic resonance imaging (MRI) has the capability to visualize the three‐dimensional structure of tissue due to high spatial resolution and deep tissue penetration without radiation.^[^ ^10^ ^]^ However, the sensitivity of MRI is relatively poor compared with positron emission tomography (PET)^[^ ^11^ ^]^ and optical imaging,^[^ ^12^ ^]^ resulting in a weak contrast resolution in distinguishing tumors from adjacent normal tissues.^[^ ^13^ ^]^ Two strategies can be used to circumvent these limitations: (1) develop a nano‐system that can load high amount of magnetic resonance contrast agents, and (2) enhance the accumulation of magnetic nanoparticles in the tumor via an active targeting process, such as by employing a receptor‐ligand interaction mechanism during the delivery process.

Currently, the paramagnetic gadolinium ion chelates are mostly used in clinical application, such as Gd‐DTPA, Gd‐DOTA, and Gd‐HPDO3A.^[^ ^14^ ^]^ These substances can increase the contrast between regions in the MRI by shortening the longitudinal relaxation time (T1) and transverse relaxation time (T2) of the surrounding water protons.^[^ ^15^ ^]^ To achieve simultaneous visualization and treatment, multifunctional nanomaterials were developed.^[^ ^16^ ^]^ For example, Luo et al. reported a dual‐functional lipid‐like nanoparticles for delivery of mRNA and MRI contrast agent.^[^ ^17^ ^]^ TT3‐Gd18 lipid nanoparticles (LNPs) showed high mRNA delivery efficiency, and T1‐weighted images of mouse legs could be clearly observed after intramuscular injection. However, the recently developed formulations are unable to maintain a balance between visualization and treatment, which not only leads to unsatisfactory diagnostic performance, but also affects the treatment effects. Therefore, it remains a huge challenge to develop a versatile system that is easy to prepare, biocompatible, and feasible to realize simultaneous diagnosis and treatment.

**SCHEME 1:**
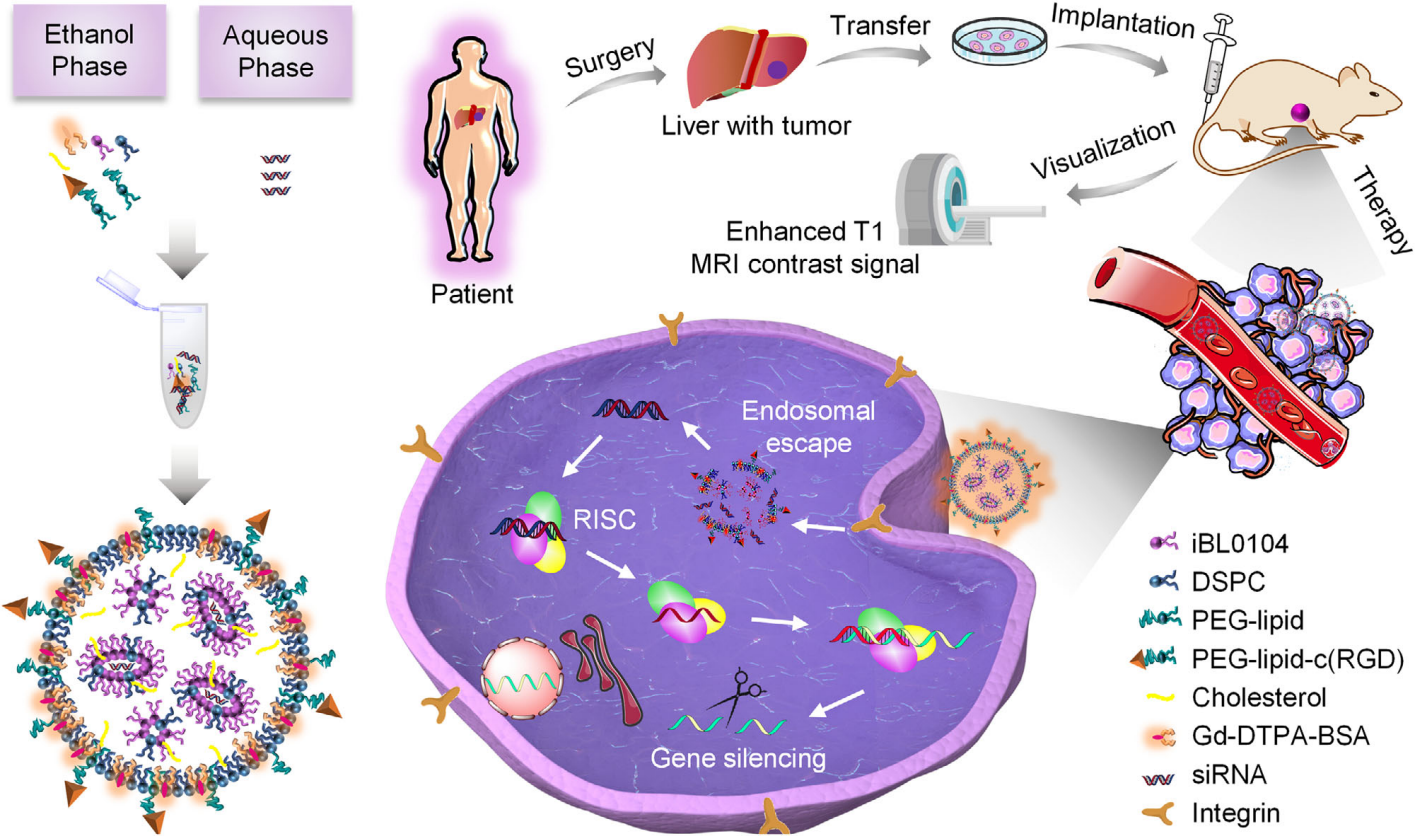
Schematic illustration of proposed ionizable lipid formulations, which enable MRI‐guided and tumor‐targeted siRNA delivery, as well as effective cancer treatment

In this study, we developed a membrane‐destabilizing ionizable lipid‐like material termed iBL0104. By employing iBL0104, DSPC, cholesterol, DSPE‐PEG_2000_ (or DMG‐PEG_2000_), contrast agent DTPA‐BSA (Gd), and siRNA, we fabricated an siRNA ionizable lipid nanoparticles (iLNPs) system (Scheme 1). Meanwhile, a tumor‐targeting cyclic RGD (cRGD) peptide could be coupled to DSPE‐PEG_2000_, conferring proposed iLNPs active targeting capability. siRNA transportation and gene silencing efficiencies of the targeted iLNPs (GARP) and iLNP without targeting modification (GAP) were carefully elucidated. Then intracellular trafficking process and in vivo biodistribution of GARP were investigated. Finally, MRI recording, in vivo antitumor effects, as well as the safety profiles were thoroughly explored in both HepG2‐luc cell line‐derived xenograft and liver cancer patient‐derived xenograft tumor models.

## 2 RESULTS AND DISCUSSION

### 2.1 Screening and optimization of iLNP formulation

iLNPs were prepared via alcohol injection method by employing self‐developed lipid‐like materials termed iBL0104 (Scheme 1). iBL0104 contains amino hydrophilic head and four hydrophobic alkyl chains, whose detailed chemical structure is shown in Figure S1. In order to make iLNPs have MRI and active targeting capabilities, DTPA‐BSA (Gd) and DSPE‐PEG_2000_‐cRGD were introduced into the nano‐systems, respectively (Scheme 1). DSPE‐PEG_2000_‐cRGD was synthesized according to previous report.^[^ ^20^ ^]^ Using the classic thiol–maleimide coupled reaction under pH 7.0, the cRGD peptide was successfully coupled with DSPE‐PEG_2000_‐MAL. MALDI–TOF–MS analysis showed that the mass peak was right‐shifted 2400 Da after the coupling process (Figure S2).

**FIGURE 1:**
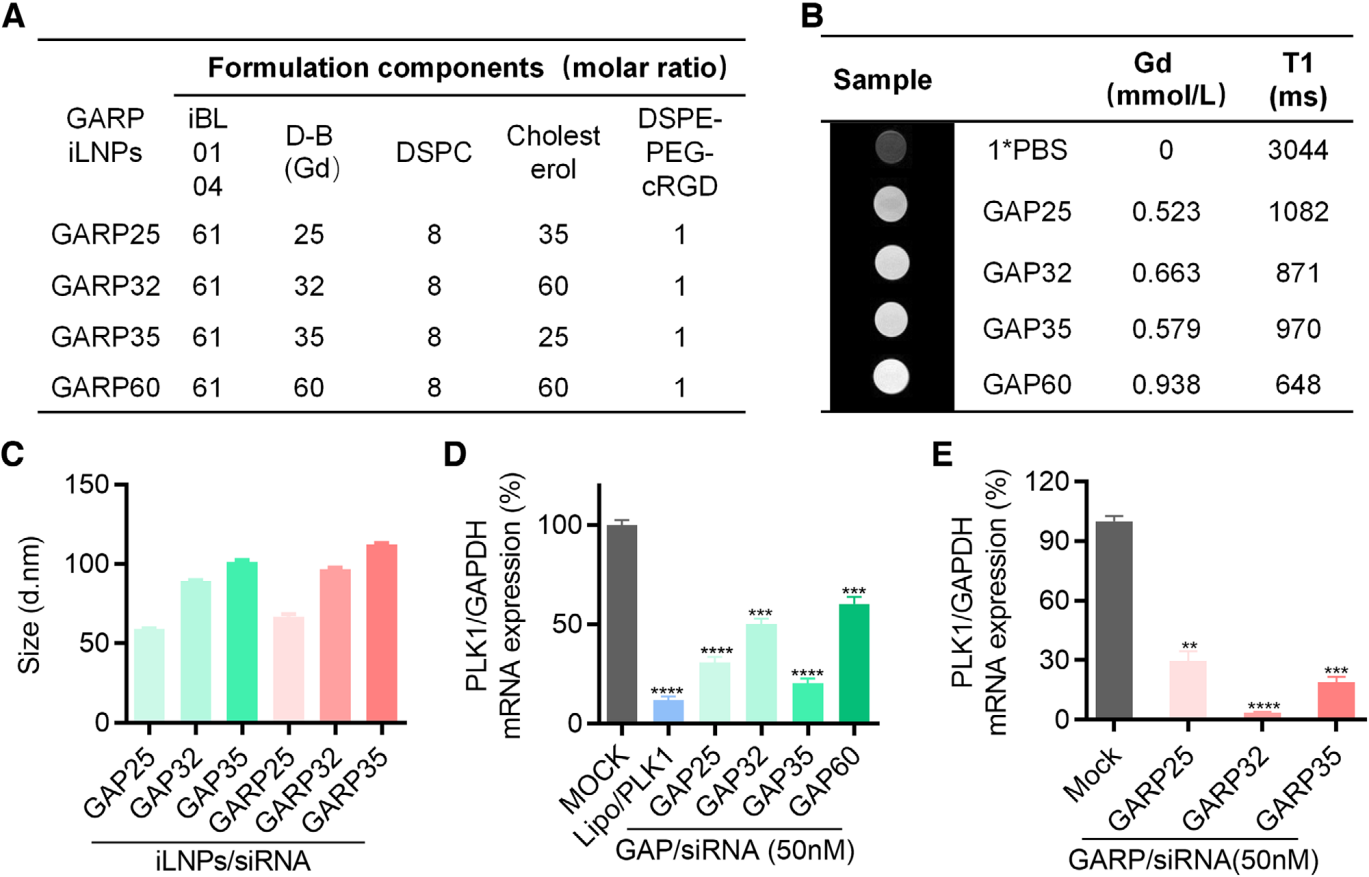
Formulation screening by evaluating the MRI effect and gene silencing activity in vitro. (A) GARP iLNP formulations prepared with various molar ratios of iBL0104, D‐B(Gd), DSPC, cholesterol, and DSPE‐PEG‐cRGD. D‐B(Gd) is short for DTPA‐BSA (Gd). (B) The imaging effects of GAP iLNPs with different amounts of Gd agent. (C) Particle sizes of various GAP/siRNA and GARP/siRNA complexes as detected by DLS. (D and E) Relative PLK1 mRNA expression in HepG2‐luc cells after treated with GAP iLNPs (D) and GARP iLNPs (E), respectively. **P < 0.01; ***P < 0.001, ****P < 0.0001

In order to establish a formulation with excellent balance and performance on MRI effect and siRNA transfection, GARP iLNPs and GAP iLNPs with various molar ratios of DTPA‐BSA (Gd) were engineered (Figure 1A; Figure S3). The MRI effect of GAP iLNPs, as shown in Figure 1B, revealed that the imaging quality enhanced as the amount of DTPA‐BSA (Gd) increased. GAP32, GAP35, and GAP60 all exhibited ideal contrast effects compared with PBS. Physicochemical properties of all iLNPs including particle sizes, zeta potentials (Figure 1C; Figure S4) and encapsulation efficiency (Figure S5) were further characterized, which illustrated that the particle sizes of GAP/siRNA iLNPs and GARP/siRNA iLNPs ranged from 59.16 ± 0.37 nm to 112.25 ± 1.04 nm. Then we analyzed the gene inhibition efficiencies of GAP/siRNA iLNPs with different molar ratios of the lipid components by using siRNA targeting PLK1 (siPLK1). PLK1 (polo‐like kinase) has been found to play a key role in cell‐cycle progression and inhibition of PLK1 has been shown to delay spindle apparatus formation during mitosis and impede chromosome alignment at the equator during metaphase, as well as promoting apoptosis.^[^ ^21^ ^]^ In addition to having an essential role in mitosis, PLK1 has been shown to be an important regulator of the DNA damage checkpoint.^[^ ^22^ ^]^ As shown in Figure 1D, GAP35/siPLK1 iLNPs exhibit the highest gene silencing activity compared with other iLNP formulations. In addition, GARP/siPLK1 iLNPs showed the best gene silencing activity compared with other GAP/siPLK1 iLNPs (Figure 1E). In terms of the MRI and gene silencing effects of proposed formulations, GAP35 and GARP35 iLNPs were overall superior than other nanoparticles, therefore they were employed to perform following studies.

### 2.2 Exploration of GARP35 and GAP35‐mediated siRNA transfection in vitro

**FIGURE 2:**
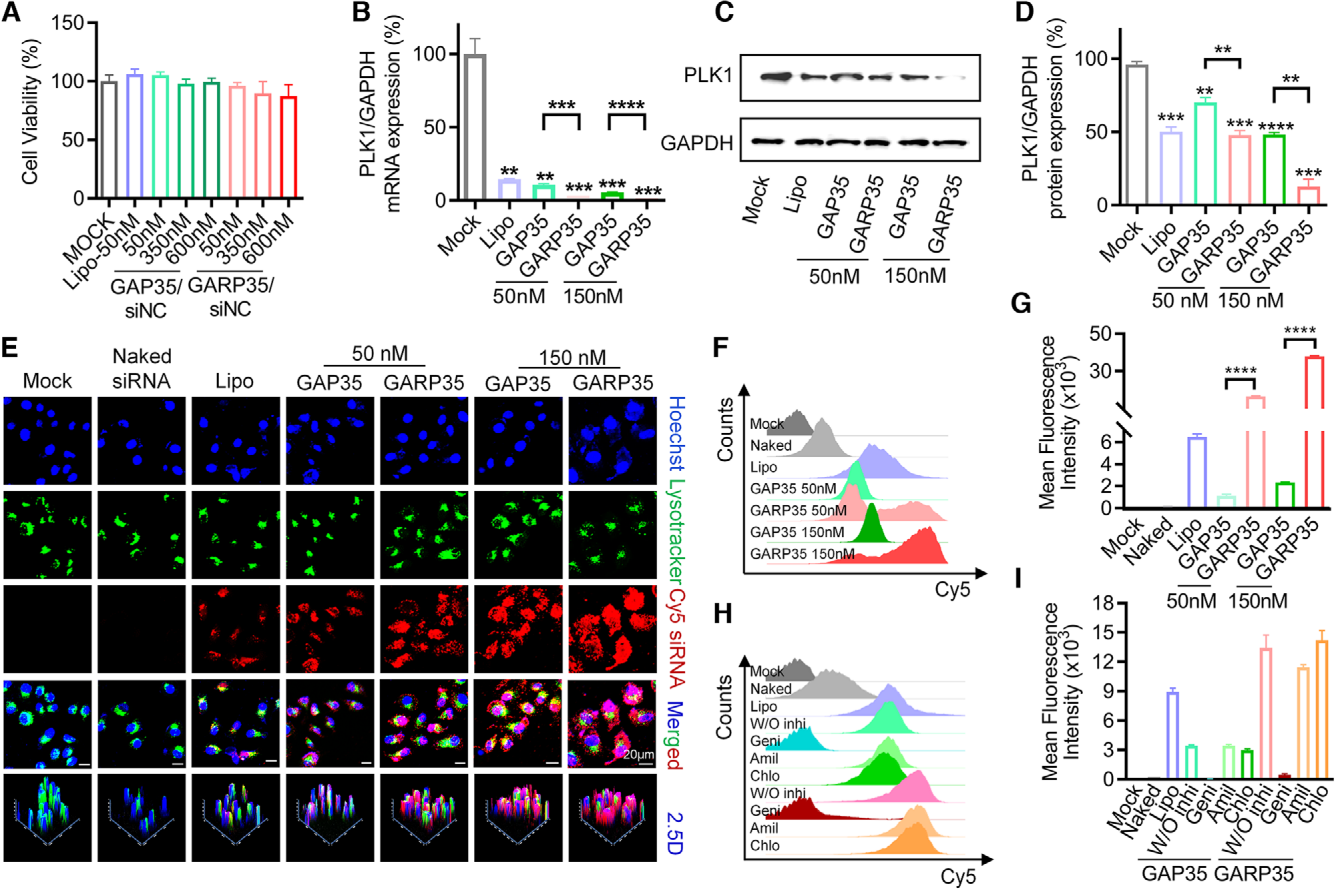
In vitro siRNA transfection mediated by GAP35 and GARP35 iLNPs on HepG2‐luc cells. (A) Viability of HepG2‐luc cells received various iLNPs treatment. (B) Relative PLK1 mRNA expression as detected by qRT‐PCR. siRNA was transfected 50 and 150 nM, respectively, for both GAP iLNPs and GARP iLNPs. (C) PLK1 protein level as analyzed by Western blotting. (D) Quantitative analysis of (C) with Image J software. (E) Confocal imaging of HepG2‐luc cells transfected with GAP35/Cy5‐siRNA and GARP35/Cy5‐siRNA for 4 h at the final siRNA concentration of 50 and 150 nM, respectively. (F) Cellular uptake of GAP35/Cy5‐siRNA and GARP35/Cy5‐siRNA formulations as recorded by fluorescence‐activated cell sorting (FACS). (G) Quantitative analysis of (F). (H) Internalization mechanism exploration of GAP35/Cy5‐siRNA and GARP35/Cy5‐siRNA complexes by using three inhibitors involved in three different pathways. The siRNA transfection concentration was 50 nM. (I) Quantitative analysis of (H). Each bar represents the mean ± SEM of three replicates. **P < 0.01; ***P < 0.001; ****P < 0.0001

MTT assay was performed to evaluate the cytotoxicity of GARP35/siNC iLNPs and GAP35/siNC iLNPs on HepG2‐luc cells (Figure 2A). Data showed that no obvious cytotoxicity was observed for all formulations, even when the siRNA transfection concentration reached to 600 nM. Then gene silencing activities of GARP35/siPLK1 iLNPs and GAP35/siPLK1 iLNPs at different transfection concentrations (50 nM and 150 nM) were further analyzed. Data showed that both GAP35/siPLK1 and GARP35/siPLK1 iLNPs exhibited significant gene silencing in vitro (Figure 2B). The inhibition efficiencies reached 89.30% and 94.49% for GAP35/siPLK1‐treated cells at 50 nM and 150 nM, respectively, and the silencing efficiencies were 96.64% and 98.74% for GARP35/siPLK1‐treated cells at 50 nM and 150 nM, respectively (Figure 2B). Lipo‐transfected siPLK1 mediated 85.30% inhibition of mRNA expression. It is worth noting that GARP35/siPLK1‐mediated mRNA knockdown was significantly higher than GAP35/siPLK1‐mediated knockdown, suggesting that cRGD decoration dramatically enhanced the internalization of the nanoparticles via RGD/integrin interaction mechanism. Examination of protein expression by Western blotting also proved these observations (Figure 2C and D).

Cell uptake efficiency and internalization pathway were explored to unveil the underlying mechanism of GAP35/siRNA and GARP35/siRNA‐mediated gene silencing. Briefly, HepG2‐luc cells were incubated with GAP35/Cy5‐siRNA and GARP35/Cy5‐siRNA for 4 h at the final siRNA concentration of 50 nM and 150 nM, respectively. Confocal imaging and quantitative analysis of Cy5‐siRNA revealed that both GAP35/Cy5‐siRNA and GARP35/Cy5‐siRNA complexes showed stronger Cy5 fluorescence intensity than Lipo/Cy5‐siRNA (Figure 2E; Figures S6 and S7). GAP35/Cy5‐siRNA and GARP35/Cy5‐siRNA complexes transfected at 150 nM displayed higher accumulation of siRNA in cells than those complexes transfected at 50 nM. GARP35 iLNPs transported larger amounts of Cy5‐siRNAs into the cells than GAP35 iLNPs (Figure 2E). In addition, flow cytometry was employed to evaluated cellular entry of proposed formulations. Both the fluorescence histogram and quantitative analysis data revealed similar observations to confocal imaging (Figure 2F and G). Cells treated with GARP35/Cy5‐siRNA iLNPs at the final siRNA concentrations of 50 and 150 nM showed higher mean fluorescence intensity (MFI) than those treated with GAP35/Cy5‐siRNA iLNPs at the same siRNA concentrations, respectively. The MFIs of the former were approximate 15 times and 16 times that of the latter, respectively. Collectively, these results demonstrated that introduction of cRGD moiety on the surface of nanoparticles enabled more effective cellular entry in targeted cancer cells than those without cRGD modification. All these observations were in line with the gene silencing results.

In order to further explore the mechanism of endocytosis, common endocytic inhibitors including amiloride (Amil), chlorpromazine (Chlo), and genistein (Geni) were used to block macropinocytosis, clathrin‐mediated endocytosis, and caveolin‐mediated endocytosis, respectively. The inhibitors were added to the cell culture medium before transfection with GAP35/Cy5‐siRNA iLNPs or GARP35/Cy5‐siRNA iLNPs. GAP35/Cy5‐siRNA and GARP35/Cy5‐siRNA groups without inhibitors (W/O inhi) were included as controls. It was observed that the cellular entry of the GAP35/Cy5‐siRNA and GARP35/Cy5‐siRNA complexes was significantly inhibited when the cells were treated with genistein (Figure 2H and I). The MFI in genistein‐treated cells was even less than 1000 (unit). Meanwhile, the MFIs of GARP35/Cy5‐siRNA groups were still higher than those of GAP35/Cy5‐siRNA groups (Figure 2I). These results were consistent with the patterns recorded in Figure 2F, highlighting the contribution of cRGD decoration. In the cells receiving the treatment of amiloride (Amil) and chlorpromazine (Chlo), the endocytosis of GAP35/Cy5‐siRNA and GARP35/Cy5‐siRNA were barely affected, compared with the cells without treating with inhibitor. This study demonstrated that caveolin‐mediated endocytosis played a dominant role in mediating internalization of GAP/siRNA and GARP/siRNA iLNPs by HepG2‐luc cells.

### 2.3 Intracellular trafficking and endosomal escape of GARP35 iLNPs

**FIGURE 3:**
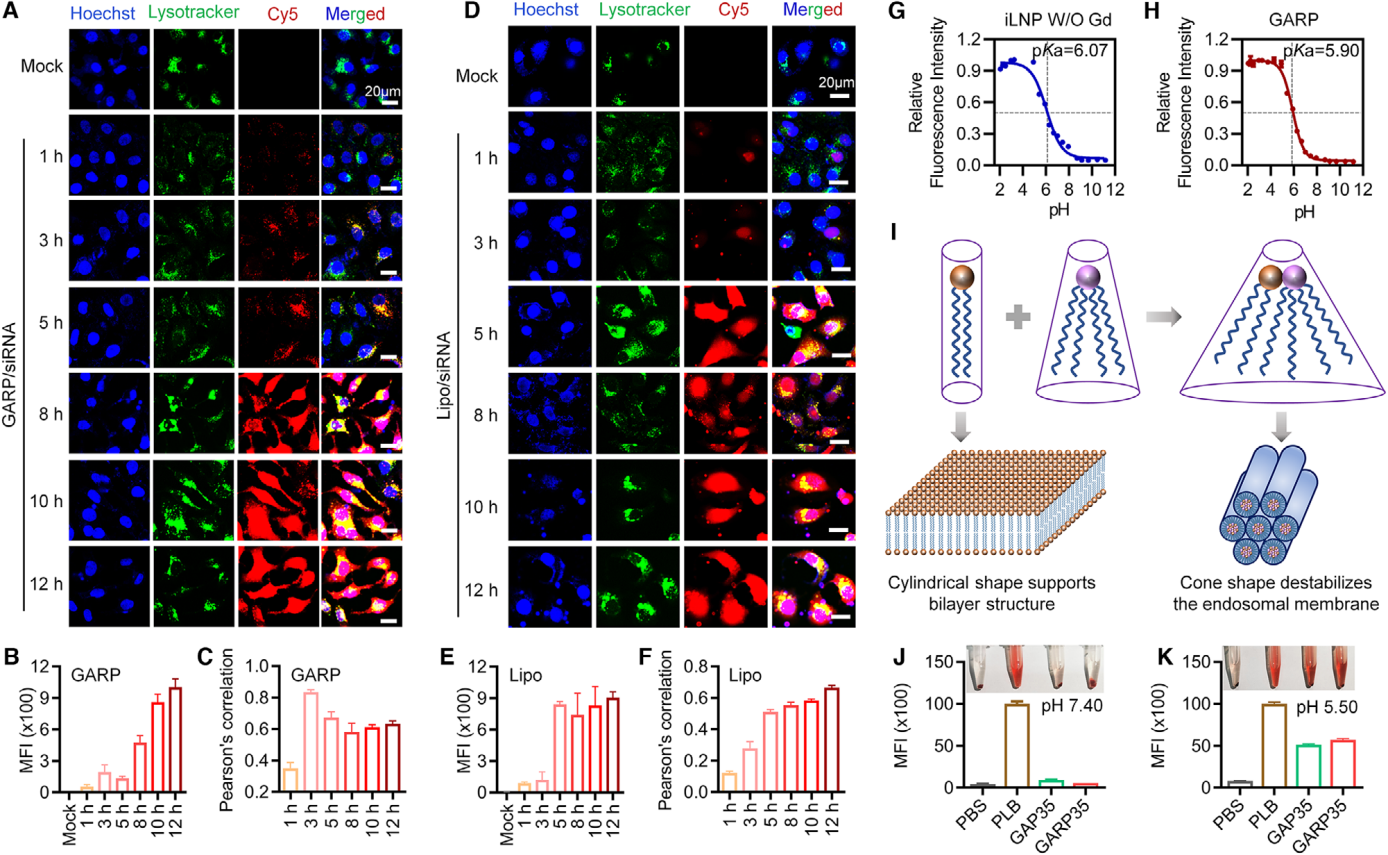
Intracellular trafficking of GARP35 iLNPs and p*K*a‐driven endosomal escape process in HepG2‐luc cells. (A–F) Confocal laser scanning microscopy (CLSM) imaging and quantitative analysis of HepG2‐luc cells transfected with GARP35/Cy5‐siRNA (A–C) and Lipo/Cy5‐siRNA (D–F) at indicated time points after transfection. (A and D) Confocal images of cells received the treatments of GARP35/Cy5‐siRNA (A) and Lipo/Cy5‐siRNA (D), respectively. (B and E) MFIs of GARP35/Cy5‐siRNA iLNPs (B) and Lipo/Cy5‐siRNA (E), respectively. (C and F) Pearson correlation analysis of (A) and (D), respectively. (G and H) p*K*a values calculated from the TNS fluorescence titration curves of iLNP W/O Gd NPs (G) and GARP NPs (H). (I) Proposed membrane destabilization mechanism of GARP iLNPs. (J and K) Results of hemolytic assay in vitro

To further investigate the intracellular trafficking process of GARP35/siRNA complexes, fluorescence signals of Cy5‐siRNA in HepG2‐Luc cells were recorded by fluorescence confocal microscopy at 1, 3, 5, 8, 10, and 12 h after transfection (Figure 3A). Lipo, a commercial cationic lipid transfection agent, was used as a control. It was suggested that intracellular Cy5 signal gradually increased with the extension of transfection time, which was consistent with the trend of quantitative analysis data shown in Figure 3B. In addition, the co‐localizations of siRNA and endosome/lysosome were analyzed by calculating the Pearson's correlation (Figure 3C). The results showed that the Pearson's correlation reached the maximum value at approximate 3 h after transfection for GARP35/siRNA iLNPs, revealing that the nanoparticles mainly located in the endosome (or lysosome) after entering the cells in the initial time range (within approximate 3 h). After that, siRNAs successfully escaped from the endosome (or lysosome), because the Pearson's correlation began to decrease at 5 h after transfection. As for Lipo, generally comparable amount of siRNA were internalized by the cells compared with GARP/siRNA complexes. However, Pearson's correlations recorded in cells treated with Lipo/siRNA continued to increase with the extension of the transfection time, suggesting that siRNA may not be able to quickly escape from the endosome (or lysosome) (Figure 3D–F).

In addition, to comprehensively unveil the underlying mechanism of GARP‐mediated rapid and effective endosomal escape, the p*K*a values of iLNPs without Gd agent (iLNP W/O Gd) and GARP iLNPs were determined by titrating with 2‐(p‐toluidino)‐6‐napthalene sulfonic acid (TNS). It was observed that their p*K*a values were 6.07 and 5.90, respectively (Figure 3G and H). It was reported that p*K*a value of lipidoid (lipid‐like material) nanoparticles must meet or exceed 5.5,^[^ ^23^ ^]^ otherwise the nanoparticles barely displayed desired *in vivo* siRNA transportation efficiency. Therefore, proposed GARP iLNPs may trigger effective endosomal escape via a membrane‐destabilization mechanism.^[^ ^7^, ^8^, ^24^ ^]^ When the lipid molecules of the endosome (or lysosome) exist independently, the anionic lipids will maintain a columnar shape (Figure 3I). When GARP iLNPs accumulate in the endosome (or lysosome), the surface protonation of GARP iLNPs in acidic environment leads to increase of positive groups and promotes the formation of ion pairs between cationic lipids and endolysosomal membrane anionic lipids such as phosphatidylserine (Figure 3I). Then ion pairs can form a conical structure, which destabilizes the endolysosomal membrane, resulting in the release of siRNA into the cytoplasm. In addition, hemolysis analysis was performed to evaluate the safety of the GAP35/siRNA and GARP35/siRNA nanocomplexes in vivo and the escape efficiency in endosomes/lysosomes. PLB was used as positive control. siRNA‐loaded GAP35/GARP35 iLNPs and red blood cells were incubated in pH 7.4 1× PBS buffer for 2 h, no obvious hemolysis was observed, which preliminarily proved that GAP35/GARP35 iLNPs had good biosecurity and could be used for animal experiments (Figure 3J). However, when the above complexes and red blood cells were co‐incubated in pH 5.5 1× PBS buffer for 2 h, significant hemolysis was observed, reflecting the high escape efficiency of the GAP35/siRNA and GARP35/siRNA iLNPs in the endosomes/lysosomes (Figure 3K).

### 2.4 In vivo distribution of GARP35 iLNPs in tumor‐bearing mice

**FIGURE 4:**
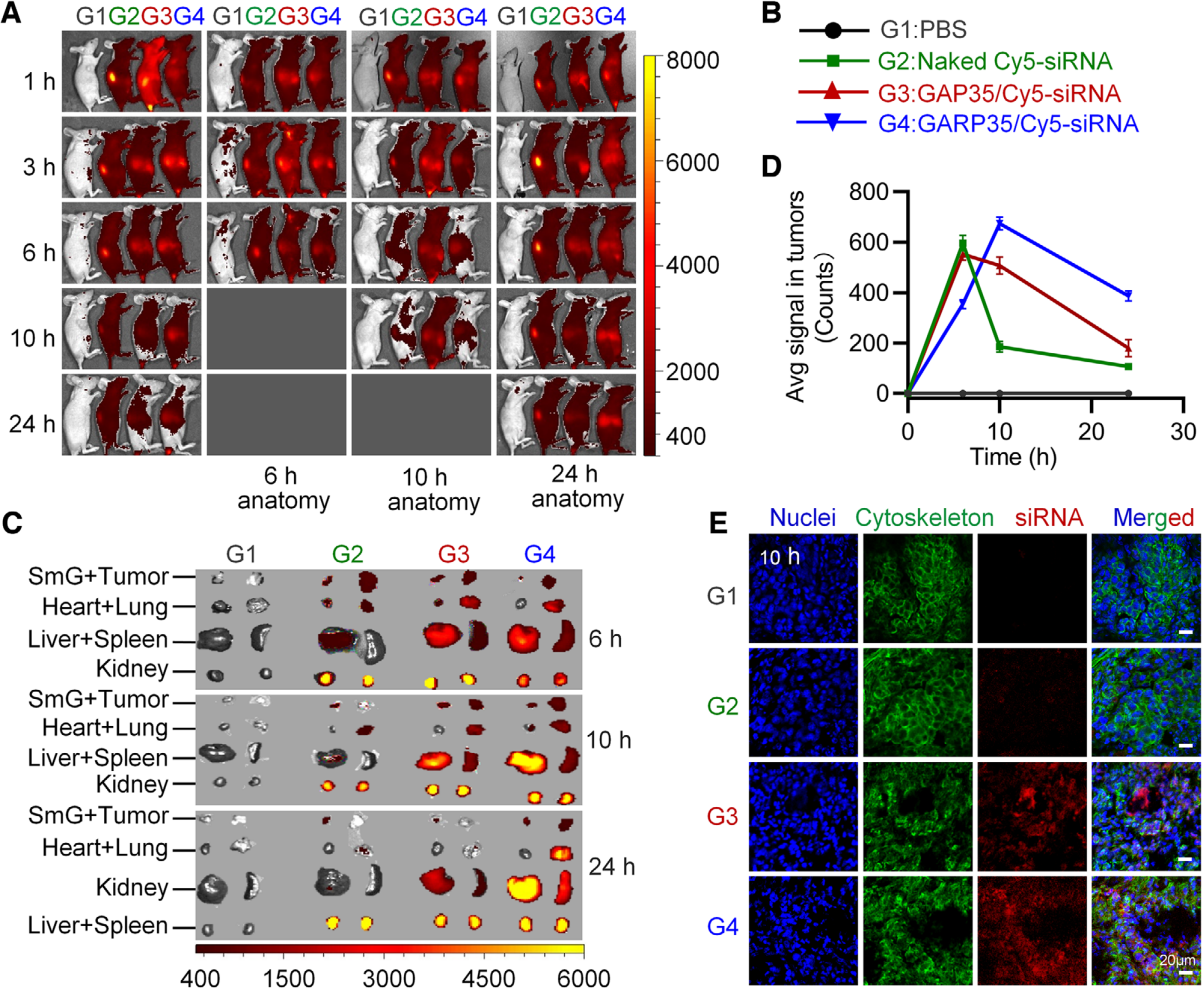
In vivo evaluation of tumor targeting behavior of GARP/siRNA iLNPs. (A) Whole body imaging at given time points after intravenous injection. (B) Grouping information. (C) Fluorescence detection of isolated organs and tumor tissues. (D) Quantitative analysis of the tumors isolated at 6, 10, and 24 h after injection. (E) Confocal observation of cryosections of tumors collected at 10 h after injection. The nucleus was stained with DAPI (blue), and cytoskeleton was stained with FITC‐labeled phalloidin (green). Scale bar, 20 µm

Encouraged by the promising transfection data in vitro, we next examined the biodistribution of GAP35/Cy5‐siRNA and GARP35/Cy5‐siRNA complexes in tumor‐bearing mice. Mice were administered with 1× PBS, Naked Cy5 siRNA, GAP35/Cy5‐siRNA, and GARP35/Cy5‐siRNA complexes via intravenous injection. The distributions of Cy5 fluorescence signal in the tumor and the whole body were detected at indicated time points post injection (Figure 4A). Following whole body imaging, the tumor tissues and main organs were isolated and re‐examined (Figure 4B), and then the tumor tissues were quantitatively analyzed (Figure 4C). Data showed that naked Cy5‐siRNA was rapidly eliminated mainly through kidney‐urine pathway,^[^ ^25^ ^]^ and the fluorescence signal in the tumor was barely visible at 10 h post injection. By contrast, an increased fluorescent signal could be detected in the tumors injected with GAP35/siRNA and GARP35/siRNA. Quantitative analysis data manifested that the fluorescence intensity in the tumors suffered GARP35/siRNA treatment was stronger than that in GAP35/siRNA‐treated tumors at 10 and 24 h after injection (Figure 4C), revealing that GARP35/siRNA iLNPs were metabolized more slowly in mice than GAP35/siRNA iLNPs. It is assumed that cRGD moiety enabled more nanoparticles being internalized by the cells in GARP35/siRNA‐treated animals than that in GAP35/siRNA‐treated mice. Furthermore, cryosections of tumor tissues were prepared and stained with DAPI (for staining nuclei) and FITC‐labeled F actin (for staining F actin and showing the rough cell outline) at 6, 10, and 24 h post‐injection. Data revealed that the Cy5 signal could be observed in GAP35/siRNA group and GARP35/siRNA group, and the signal intensities in GARP35/siRNA group were generally stronger than those in GAP35/siRNA group (Figure 4D; Figure S8). These observations were in line with the quantitative data shown in Figure 4C. Taken together, GARP iLNPs with cRGD targeting modification exhibited higher siRNA transportation efficiency than GAP iLNPs without cRGD decoration in tumor‐bearing mice, encouraging us to perform more in vivo assays to evaluate the anti‐tumor effects of GARP iLNPs.

### 2.5 Antitumor effects of GARP35 iLNPs on CDX model

**FIGURE 5:**
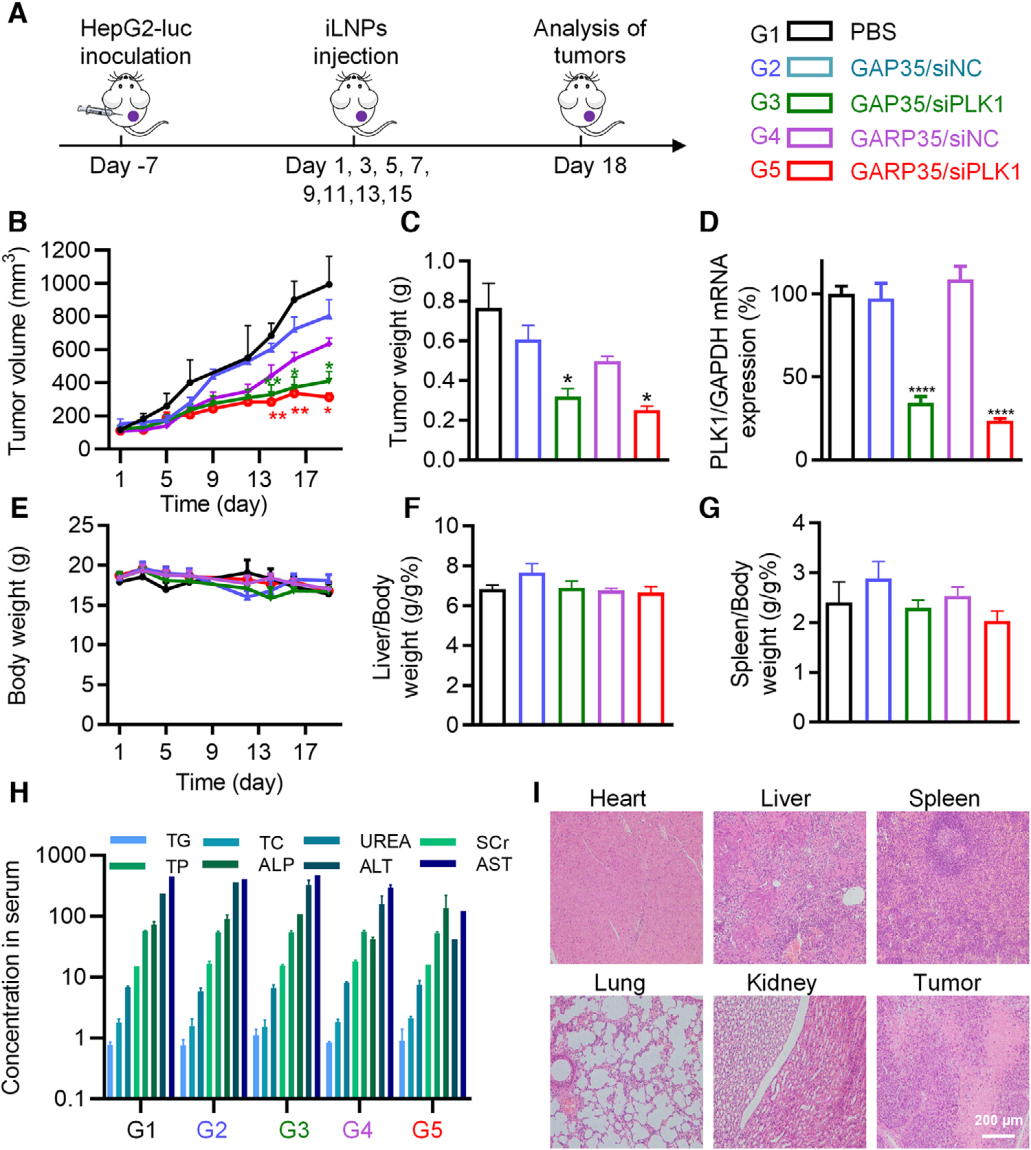
Tumor growth inhibition on HepG2‐luc cell derived xenograft (CDX) model. (A) Schematic illustration of the treatment course and animal grouping. (B) Tumor growth curves recorded in various groups. (C) Tumor weight recorded at day 18 when the animals were sacrificed. (D) PLK1 mRNA expression in tumors. (E) Body weight monitored during the treatment course. (F and G) Organ coefficients of the liver (F) and the spleen (G) at the end of the experiment (day 18). (H) Serum biochemistry parameters measured at the end of the experiment. (I) H&E staining of the heart, liver, spleen, lung, kidney and tumor in GARP/siPLK1 group. Data are shown as mean ± SEM (*n* = 6). *P < 0.05, **P < 0.01, ****P < 0.0001

Encouraged by the performances of GARP35 iLNPs on targeted delivering siRNA in vitro and in vivo, we further evaluated its antitumor effect in HepG2‐luc cell line‐derived xenograft murine models. Once the tumor volumes reached 100 to 200 mm^3^, mice were randomly divided to five groups with six animals per group, and administered with following formulations: (1) PBS, (2) GAP35/siNC, (3) GAP35/siPLK1, (4) GARP35/siNC, and (5) GARP35/siPLK1, respectively (Figure 5A). It was observed that the tumor growth was significantly inhibited for the mice receiving the treatment of GAP35/siPLK1 and GARP35/siPLK1 (Figure 5B). The inhibition efficiency in GARP35/siPLK1 group was higher than that in GAP35/siPLK1 group (*P* < 0.05). Integrin (α_v_β_3_) is usually expressed at extremely low levels in normal cells, but can be highly expressed in many types of cancer cells^[^ ^26^ ^]^ including HepG2‐luc liver cancer cells.^[^ ^27^ ^]^ Hence, cRGD‐modified GARP35 formulation could interact with HepG2‐luc more efficiently than GAP35 nanoparticle without cRGD decoration, which boosted the anticancer treatment efficacy. There was no significant difference among the groups of PBS, GAP35/siNC, and GARP35/siNC (Figure 5B).

At the end of treatment, mice were sacrificed and tumor tissues were isolated and weighed. Data showed that the weights of tumor tissues collected from the mice treated with GARP35/siPLK1 or GAP35/siPLK1 were dramatically lower than those recorded in the groups of PBS, GAP35/siNC, and GARP35/siNC (Figure 5C). To verify the knockdown of the target gene expression, mRNA level of PLK1 in tumors was analyzed by qPCR (Figure 5D). It was suggested that the expression of PLK1 mRNA in GARP35/siPLK1 group and GAP35/siPLK1 group were remarkably reduced, with an inhibition efficiency of 76% and 66%, respectively. In addition, during the entire experimental period, the body weights of all animals, as well as the organ coefficients of the liver and spleen, did not change significantly (Figure 5E–G). Moreover, eight serum biochemistry parameters including triglyceride (TG), total cholesterol (TC), serum creatinine (SCr), blood urea nitrogen (UREA), total protein (TP), alkaline phosphatase (ALP), alanine aminotransferase (ALT), and aspartate transaminase (AST) were examined, suggesting that GAP35/siPLK1 and GARP35/siPLK1 were well tolerated by the animals (Figure 5H). H&E staining results also demonstrated that GARP35/siPLK1 and other groups showed no pathologically significant change in the major organs of the mice (Figure 5I and S5). While cell apoptosis could be observed in tumor sections prepared from the groups of GAP35/siPLK1 and GARP35/siPLK1 (Figure 5I and S9).

### 2.6 MRI and antitumor therapy of iLNP/siRNA on PDX model

**FIGURE 6:**
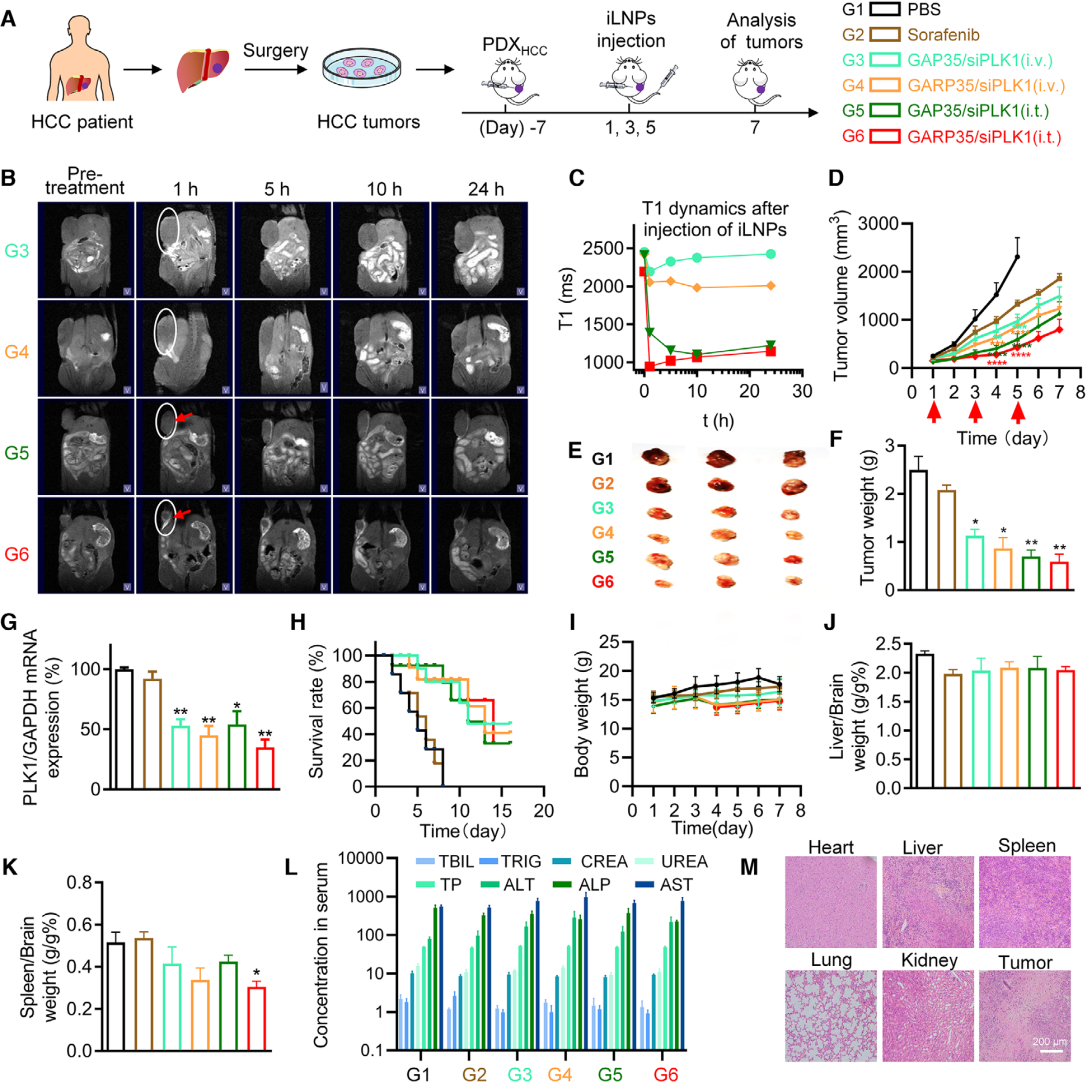
MRI and anticancer effects of iLNP formulations in PDX model. (A) Schematic illustration of the treatment protocol and grouping information. (B) MRI images acquired before administration and at 1, 5, 10, and 24 h after intravenous or intratumoral administration of lipid/siRNA formulations. The tumors were marked with white ellipse, and intratumorally treated tumors were indicated with red arrows. (C) Quantitative analysis of the tumor tissues shown in (B). (D) Tumor growth inhibition after treated with various formulations in PDX model. (E) Optical images of the tumors isolated on day 5. (F) Average tumor weights recorded on day 5. (G) Expression of PLK1 mRNA in tumor tissues. (H) Survival curves of the tumor‐bearing mice. (I) Body weights of the mice during the treatment course. (J and K) Organ coefficients of the liver (J) and the spleen (K), which were calculated by dividing the weight of the liver to the weight of the brain, and the weight of the spleen to the weight of the brain, respectively. (L) Serum biochemistry parameters examined at the end of experiment. (M) H&E staining of the main organs and the tumor tissue in the G4 group. Data were shown as the mean ± SEM. *P < 0.05, **P < 0.01, ***P < 0.001, ****P < 0.0001 vs PBS group

Visualization of the tumors and therapeutic agents is of great significance for precise cancer treatment in clinical practice. Accordingly, we further evaluated the accumulation of proposed lipid formulations and their treatment effects in liver cancer patient‐derived xenograft (PDX) murine model (Figure 6A). In this study, six groups of mice with nine animals per group were treated with (1) PBS, (2) Sorafenib, (3) GAP35/siPLK1 (i.v.), (4) GARP35/siPLK1 (i.v.), (5) GAP35/siPLK1 (i.t.), and (6) GARP35/siPLK1 (i.t.), respectively. “i.v.” and “i.t.” represent intravenous injection and intratumoral injection, respectively. Animals in groups 3, 4, 5, and 6 were used to perform MRI before administration and after receiving the first dose of lipid formulations (Figure 6B). For animals receiving intravenous injection of GAP35/siPLK1or GARP35/siPLK1, they exhibited enhanced MRI signals at the margin of tumor tissue 1 h after injection. The average T1 weighted value of the mice injected with GAP35/siPLK1 reached the maximum values at 1 h, as indicated by the MRI and quantitative analysis result (Figure 6B and C). The average T1 weighted value of the mice injected with GARP35/PLK1 NPs increased slightly with time extension (Figure 6B and C). The intratumoral injection groups also showed enhanced MRI signal at the injection site (Figure 6B, as the red arrow indicated). Quantitative analysis data showed that the lipid complexes gradually spread in tumor tissue. The diffusion rate of GARP35/PLK1 group was faster than that of GAP35/siPLK1 group (Figure 6B and C). It was also observed that GARP35/siRNA accumulated at the tumor sites at higher levels compared with GAP35/siRNA for both tail vein injection and intratumoral injection groups (Figure 6C), leading to a decrease in the MRI signal intensity after 5 h post‐injection for GARP35/siRNA. These results indicated that the cRGD conjugation on the lipid nanoparticle enhanced the tumor targeting capability of proposed formulation in vivo.

Consequently, two additional doses of lipid formulations were administered to the animals every two days (Figure 6A and D). The body weights and tumor volumes were recorded every day until the end of the experiment. It was revealed that the tumors in PBS group grew quickly, as the average tumor volume reached over 2000 mm^3^ at day 5 (Figure 6D). In contrast, tumors grew substantially more slowly in the mice treated with GARP35/siPLK1, regardless tail vein injection or intratumoral injection. In addition, tumor suppression effect in intratumoral injection group is overall superior to that in intravenous injection group, and the tumor growth rate in the mice treated with GARP35/siPLK1 was slower than that in the mice treated with GAP35/siPLK1.

When the average tumor volume in the PBS group reached approximate 2000 mm^3^, three animals were randomly selected and sacrificed in each group. The tumor tissues, as well as the main organs, were isolated and weighted. Optical images of the isolated tumors were also acquired, and the expression of PLK1 in tumor tissues were determined by qRT‐PCR. Data showed that the tumors collected from iLNP/siPLK1‐treated mice were remarkably smaller than those collected from PBS group (Figure 6E). Similar profiles were observed from the tumor weighting data (Figure 6F). Tumor growth curve, optical image and tumor weight data revealed that orally‐dosed Sorafenib, a small molecule drug used for liver cancer treatment in clinical practice, exhibited slight tumor inhibition efficiency in this patient derived model (Figure 6D–F). PLK1 mRNA expression were significantly repressed in animals receiving siRNA treatment, regardless the administration route or targeting decoration (Figure 6G).

Furthermore, six animals in each group were used to analyze the survival situation after treating with various formulations, which suggested that the survival time of the animals treated with lipid/siRNA formulations (groups 3, 4, 5, and 6) was dramatically extended compared with PBS group (Figure 6H). In detail, the median survival time for mice treated with PBS was 5 days, and that for Sorafenib‐treated animals slightly increased to 6 days. Both GAP35/siPLK1 (i.v.) and GAP35/siPLK1 (i.t.) exhibited a median survival time of 11 days. Treatments of GARP35/siPLK1 iLNPs (i.v.) and GARP35/PLK1 iLNPs (i.t.) extended the median survival time of the mice to 13 days and 14 days, respectively.

In addition, there were no obvious changes in body weight of the mice during the entire treatment period (Figure 6I). No significant difference was observed for the organ coefficient of the liver (the weight ratio of the liver to the brain) (Figure 6J), while the organ coefficient of the spleen (the weight ratio of the spleen to the brain) in GARP/siPLK1 group was lower than that in PBS group (Figure 6K). It is well known that the weight of the spleen in immunodeficient BALB/c nude mouse typically is higher than that in normal mouse. Hence, it was assumed that the health situation was ameliorated after treating with GARP/siPLK1, resulting in reduction of the spleen weight. Furthermore, blood specimens and the major organs of the mice were applied for serum biochemical analysis and histopathological examination, respectively (Figure 6L and m; Figure S9). Data showed that there were neither obvious changes in biomarkers of liver or kidney function nor noticeable histopathological changes in the major organs in all groups of animals.

## 3 CONCLUSION

In this study, we designed and developed two paramagnetic lipid/siRNA formulations, termed GAP35 and GARP35, by employing DSPC, cholesterol, PEGylated lipid, contrast agent DTPA‐BSA (Gd), and ionizable lipid‐like material termed iBL0104. cRGD was introduced in GARP35, while no cRGD was used in GAP35. Compared with GAP35/siRNA, GARP/siRNA exhibited enhanced siRNA transfection efficiency, higher gene silencing activity, better tumor‐targeting capability, and more effective anticancer effects both in vitro and in tumor‐bearing mice. More importantly, the p*K*a values of iLNP without Gd agent and GARP35 complexes were 6.07 and 5.90, respectively, which could destabilize the endosomal membrane by interacting with anionic lipids in the membrane. This effect significantly enhanced the endosome escape efficiency after internalization in targeted cells. In addition, the Gd‐lipid contrast agents (DTPA‐BSA (Gd)) confers proposed nano‐system the capability of real‐time visualization in vivo. Therefore, this study provides an excellent example for developing MRI‐guided siRNA delivery system and cancer treatment modality.

## 4 EXPERIMENTAL SECTION

### 4.1 Materials

1,2‐Distearoyl‐*sn*‐glycero‐3‐phosphoethanolamine‐N‐[methoxy (polyethylene glycol)‐2000] (DSPE‐PEG_2000_) and 1,2‐distearoyl‐*sn*‐glycero‐3‐phosphoethanolamine‐N‐[maleimide (polyethylene glycol)‐2000] (DSPE‐PEG_2000_‐MAL) were obtained from Nanocs Laysan Co. Ltd., USA. Cholesterol, 1,2‐distearoyl‐*sn*‐glycero‐3‐phosphocholine (DSPC), (diethylenetriaminepentaacetic acid)‐bis(stearylamide)‐gadolinium salt (DTPA‐BSA (Gd)), chlorpromazine hydrochloride, genistein and adriamycin hydrochloride, dimethyl sulfoxide (DMSO), 3‐[4,5‐dimethylthiazol‐2‐yl]‐2,5‐diphenyltetrazolium bromide (MTT), LysoTracker Green DND‐26, and Hoechst 33342 were purchased from Sigma‐Aldrich. The cyclic octapeptide c(Gly‐Arg‐Gly‐Asp‐Ser‐Pro‐Lys‐Cys) (cGRGDSPKC) was obtained from Shanghai Top‐peptide Biotechnology Co., Ltd. (China). Dulbecco's modified Eagle's medium (DMEM), Opti‐MEM, trypsin, penicilin‐streptomycin, fetal bovine serum (FBS), Lipofectamine 2000 (Lipo), and SYBR^®^ Gold Nucleic Acid Gel Stain were purchased from Invitrogen Corporation (Carlsbad, CA). Agarose was purchased from GEN TECH (Hong Kong, China). All siRNAs used in this study, including Cy5‐labeled siRNA (Cy5‐siRNA), negative controlled siRNA (siNC), and PLK1‐against siRNA (siPLK1), were provided by Suzhou Ribo Life Science Co., Ltd. (Jiangsu, China). Their sequences were as follows: siNC, sense: 5′‐CCUUGAGGCAUACUUCAAAdTdT‐3′, antisense: 5′‐UUUGAAGUAUGCCUCAAGGdTdT‐3′; siPLK1, sense strand: 5′‐UGAAGAAGAUCACCCUCCUUAdTdT‐3′, antisense strand: 5′‐UAAGGAGGGUGAUCUU CUUCAdTdT‐3′. Cy5‐siRNA is modified with Cy5 at the 5′ end of the sense chain of siNC. Chemical modifications were introduced in these siRNAs at certain sites of both strands. Ionizable lipid of iBL0104 was designed and synthesized in house.

### 4.2 Preparation of siRNA‐loaded GAP and GARP iLNPs

The GAP/siRNA iLNPs were formulated using iBL0104, cholesterol, DTPA‐BSA‐Gadolinium (DTPA‐BSA (Gd)), DSPC, DMG‐PEG_2000_, and siRNA (siNC, siPLK1 or Cy5‐siRNA). Briefly, the iBL0104, cholesterol, DTPA‐BSA (Gd), DSPC, and DMG‐PEG_2000_ were dissolved in ethanol and mixed at a molar ratio of 61: x: y: 8: 1 (x = 35, 60, 25, 60, y = 25, 32, 35, 60). Then, the organic phase was injected into a threefold volume of 50 mM pH 4.0 sodium citrate buffer solution at high speed to form iLNPs. Subsequently, the GAP iLNPs were incubated with an equal volume of siRNA for encapsulation at 50°C for 30 min. The weight ratio between the total lipids and siRNA was approximate 15:1. Finally, all formulations were dialyzed in 1× PBS for at least 2 h.

In addition, the GARP/siRNA iLNPs decorating with targeting moiety of c(GRGDSPKC) (cRGD) were prepared by replacing DMG‐PEG_2000_ with DSPE‐PEG_2000_‐cRGD. To prepare GARP iLNPs, the DSPE‐PEG_2000_‐cRGD was synthesized first. cRGD peptide and DSPE‐PEG_2000_‐MAL (wt/wt, 1:5) were dissolved in 100 mM HEPES buffer (pH 7.0), and then stirred for 48 h at 4°C. The final product was dialyzed in deionized water for 24 h by using 2 kDa dialysis bags. The identification of DSPE‐PEG_2000_‐cRGD was performed by using matrix‐assisted laser desorption/ionization time‐of‐flight mass spectrometry (MALDI‐TOF‐MS, Bruker Daltonics, USA). Then, iBL0104, cholesterol, DTPA‐BSA (Gd), DSPC, and DSPE‐PEG_2000_‐cRGD were used to prepare GARP iLNPs.

### 4.3 Dynamic Light Scattering Assay

To characterize the physicochemical properties of all iLNPs, GARP/siRNA iLNPs and GAP/siRNA iLNPs were prepared in advance. The particle sizes and zeta potentials were measured through Dynamic Light Scattering (DLS) (Zetasizer Nano ZS, Malvern, U.K.) at room temperature at an incident wavelength of 677 nm and an incident angle of 90°. The stabilities of particle sizes and zeta potentials within 2 weeks were also tested.

### 4.4 MRI in vitro

In order to evaluate the contrast effect of GARP iLNPs, MRI was performed with 7.0‐T animal MRI instrument (Bruker, Germany). T1 weighted images with different concentrations of GAP iLNPs dissolved in PBS were obtained, wherein, repetition time (TR) = 3000 ms, echo time (TE) = 40 ms.

### 4.5 Cytotoxicity analysis

MTT assay was conducted to evaluate the cytotoxicity of GARP iLNPs on cells. When HepG2‐luc cells were in the logarithmic growth phase, 10 000 cells were seeded into a 96‐well plate with 100 µL cell suspension per well and incubated for 24 h. Then the cells were transfected with siNC‐loaded GARP iLNPs at a lipid molar ratio of 61: 25: 35: 8: 1 (iBL0104: cholesterol: DTPA‐BSA (Gd): DSPC: DSPE‐PEG_2000_‐cRGD) (GARP35 iLNPs) and siNC‐loaded GAP iLNPs at a lipid molar ratio of 61: 25:35:8:1 (iBL0104: cholesterol: DTPA‐BSA (Gd): DSPC: DMG‐PEG_2000_) (GAP35 iLNPs), at the final siRNA concentration of 50 nM, 350 and 600 nM, respectively. siNC complexed with lipofectamine 2000 (Lipo) was used as the control group. Lipo/siRNA formulation was prepared according to standard manufacturer's protocol but fixed the weight/volume ratio of siRNA to Lipo at 1 µg/3 µL. Untreated cells were used as negative control (Mock). GARP35/siRNA iLNPs and GAP35/siRNA iLNPs were transfected into fresh complete DMEM containing 10% FBS for 24 h. Then, all the mediums in the plates were discarded, 95 µL fresh DMEM and 5 µL MTT (5 mg/mL) were added to each well and incubated at 37°C for 4 h. After the formazan crystals were dissolved completely, the absorbance was detected at 540 nm with a reference wavelength of 650 nm by Multi‐Mode Microplate Reader and cell viability was calculated according to the following equation:

(1) $$\mathrm{Cell}\;\mathrm{viability}\;\left(\%\right)=\frac{OD_{540\left(\mathrm{Sample}\right)}-OD_{650\left(\mathrm{Sample}\right)}}{OD_{540\left(\mathrm{Mock}\right)}-OD_{650\left(\mathrm{Mock}\right)}}\times 100$$

### 4.6 Real‐time PCR

Quantitative real‐time PCR (qRT‐PCR) was performed to assess the gene silencing efficiency. HepG2‐luc cells were seeded in six‐well plates at a density of 2 × 10^5^ cells per well. After 24 h, cells were transfected with GARP35/siPLK1 iLNPs and GAP35/siPLK1 iLNPs at the final siRNA concentration of 50 nM and 150 nM for 4 h. Total RNA was extracted and reversely transcribed using TRIzol Reagent (vazyme, Nanjing, China) and Reverse Transcription Kit (vazyme, Nanjing, China) according to standard manufacturer's instructions, respectively. Then, cDNA was quantified by qRT‐PCR system using SYBR Green PCR Master Mix. GAPDH (glyceraldehyde 3‐phosphate dehydrogenase) gene was used as the internal control.

### 4.7 Western blot

PLK1 protein expressions on HepG2‐luc cells were determined by western blotting. Cells are treated according the protocol the same with that in real‐time PCR assay. Whole cell proteins were extracted using 1× passive lysis buffer (PLB) with protease inhibitors (10 000×). The concentrations of protein were determined using BCA protein assay kit (Lot#CW0014, CWBIO). Sixty micrograms of protein were loaded and separated by SDS‐PAGE, followed by transferring to nitrocellulose membrane (NC) for blotting. The membrane was blocked with 5% BSA buffer for 1 h at room temperature, incubated with primary mouse monoclonal anti‐PLK1 antibody (1:1000, ab30394, Cell Signaling Technology, USA) overnight at 4°C, and incubated with secondary horseradish peroxidase‐conjugated antibody (1: 3000, ZSJQB Co., Ltd. China) for 1 h at room temperature. The blots were recorded and analyzed using chemiluminescence imaging system (Bio‐Rad, Bossier City, LA).

### 4.8 Subcellular localization and endosomal escape

To study the subcellular localization of siRNA‐loaded GARP iLNPs, the HepG2‐luc cells were seeded into 35 mm dishes at a density of 2 × 10^5^ cells per well. After 24 h, cells were transfected with Cy5‐siRNA‐loaded GAP35 iLNPs and GARP35 iLNPs at the final siRNA concentrations of 50 and 150 nM, respectively. Four hours later, the cells were washed three times with 1× PBS and stained with 1 µL of Hoechst 33342 solution (1 mg/mL in PBS, for staining nucleus) and 0.3 µL of LysoTracker Green (1: 3000 in PBS, for staining endosome and lysosome). After staining for 30 min, cells were washed twice with 1× PBS and then recorded with Zeiss confocal microscope (LSM700, Carl Zeiss, Germany).

In order to analyze the co‐localization of siRNA‐loaded GARP iLNPs with the endosomes or lysosomes at different transfection time points, HepG2‐luc cells were plated into 35 mm dishes at a density of 2 × 10^5^ cells per well. After 24 h, the siRNA‐loaded GARP iLNPs at the final siRNA concentration of 50 nM was added to the dishes. Lipo/Cy5‐siRNA NPs served as a control group. Then the cells were stained and imaged according to above‐mentioned protocol at 1, 3, 5, 8, 10, and 12 h after transfection.

### 4.9 p*K*a determination of GARP iLNPs

To characterize the ionizable properties of GARP nanoparticles, 1 mL of GARP nanoparticles (total lipid concentration: 2 µM) and 1 mL of nanoparticles without DTPA‐BSA(Gd) (iLNP W/O Gd) (total lipid concentration: 6 µM) were prepared. The lipid molar ratio of “iLNP W/O Gd” was 61:60:8:1 (iBL0104: cholesterol: DSPC: DMG‐PEG_2000_). First of all, 100 mM HEPES (4‐(2‐hydroxyethyl)‐1‐piperazineethanesulfonic acid) buffer, 100 mM MES, 100 mM ammonium acetate, 1300 mM NaCl, and 100 µM TNS were prepared as stock solution in distilled water. Then a series of nanoparticle solutions at different pH were prepared. These solutions contain 10 mM HEPES, 10 mM MES, 10 mM ammonium acetate, 130 mM NaCl, 80 µM GARP NPs, or 80 µM iLNP W/O Gd. Subsequently, 99 µL of nanoparticle solution and 1 µL of TNS stock solution were added to the 96‐well black opaque plates and the fluorescence value was detected at an excitation wavelength of 321 nm and an emission wavelength of 445 nm. Finally, the p*K*a of GARP NPs and iLNPs W/O Gd were calculated by fitting the Henderson–Hasselbach equation (GraphPad Prism v.8).

### 4.10 Internalization and intracellular trafficking of iLNPs

To analyze the internalization behaviors of GAP35/siRNA iLNPs and GARP35/siRNA iLNPs on HepG2‐luc cells, cells were seeded in six‐well plates at a density of 2 × 10^5^ cells per well. After 24 h, the DMEM supplemented with 10% FBS, 1% penicillin, and streptomycin were replaced with fresh complete DMEM. Then the cells were transfected with Cy5‐siRNA‐loaded GAP35 iLNPs and GARP35 iLNPs at the final siRNA concentration of 50 or 150 nM. Free Cy5‐siRNA was used as a negative control. After 4 h, cells were digested with 0.25% trypsin and washed three times with 1 mL of cold 1× PBS, followed by suspending in 400 µL 1× PBS and detecting by flow cytometry (Becton Dickinson, San Jose, CA, USA).

To elucidate the endocytosis mechanism of GARP iLNPs in cells, three inhibitors of amiloride (Amil), chlorpromazine (Chlo), and genistein (Geni) were employed to block macropinocytosis, clathrin‐mediated endocytosis and caveolin‐mediated endocytosis, respectively.^[^ ^18^ ^]^ Therefore, three pharmacological inhibitor solutions, including 100 µM amiloride‐HCl, 30 μM chlorpromazine‐HCl, and 1 mM genistein were used to treat HepG2‐luc cells for 0.5 h before incubation with iLNP formulations. Here, inhibitor‐free cells served as a positive control, and 4 h later, cells were analyzed by flow cytometry.^[^ ^19^ ^]^

### 4.11 Hemolysis assay

Hemolysis test was performed to evaluate the safety and escape effect in endosomes/lysosomes of siRNA‐loaded GAP/GARP iLNPs. Briefly, 400 μL fresh mouse blood was added to two anticoagulation tubes and centrifuged at 10 000 g for 5 min at 4°C. Each tube of cell pellet was resuspended with 2 mL pH 7.4 1× PBS buffer and 2 mL pH 5.5 1× PBS buffer, respectively. Then 200 µL cell suspension were incubated with GAP35/siNC or GARP35/siNC nanocomplexes with a total lipid concentration of 50 µg/mL for 2 h at 37°C. After incubation, the red blood cells were centrifuged at 12 000 × *g* for 5 min, and then all tubes were photographed. 200 µL supernatant containing lysed erythrocytes in each tube was transferred to a 96‐well transparent plate and absorbance was measured at 543 nm.

### 4.12 Animals

Animals used in this study were purchased from Vital River Laboratory Animal Technology and maintained in Peking University Laboratory Animal Center (an AAALAC‐accredited and specific‐pathogen‐free [SPF] experimental animal facility). All procedures involving experimental animals were performed in accordance with protocols approved by the Institutional Animal Care and Use Committee (IACUC) of Peking University. The approval number is “IMM‐LiangZC‐4.”

### 4.13 Tumor‐targeted accumulation of GARP iLNPs in vivo

To assess the tumor‐targeting properties of GARP nanoparticles in mice, HepG2‐luc cells (5 × 10^6^ cells) were suspended in 1× PBS (100 µL) and subcutaneously injected in right axillary fossa of female BALB/c nude mice weighting approximately 20 g. When the tumors grew to about 1000 mm^3^, mice were randomly divided into four groups, and received the treatments of 1× PBS, Naked Cy5‐siRNA, GAP35/Cy5‐siRNA, and GARP35/Cy5‐siRNA, respectively. Testing samples were administered via tail vein injection and the dose of siRNA was 1 mg/kg. Cy5 fluorescence signals in mice were detected by Kodak in vivo imaging system at 1, 3, 6, 10, and 24 h post injection, respectively (Kodak In‐Vivo Imaging System FX Pro, Care stream Health, USA). One animal in each group was sacrificed at 6, 10, and 24 h post injection to examine the isolated tumors and main organs.

### 4.14 Tumor suppression in cell line‐derived xenograft (CDX) murine model

To evaluate tumor growth inhibition efficiency of GAP35 iLNPs and GARP35 iLNPs in vivo, tumor‐bearing female BALB/c nude mice were prepared by injecting HepG2‐luc cells. When the tumor volume reached 100–150 mm^3^, mice were randomly divided into five groups and treated with (1) PBS, (2) GAP35/siNC, (3) GARP35/siNC, (4) GAP35/siPLK1, and (5) GARP35/siPLK1, respectively. The testing formulations were administered by intratumoral injection every other day at the siRNA dose of 1 mg/kg. Body weights were monitored during the entire experiment. Tumor volumes were recorded before dosing and calculated with the following formula: tumor volume (mm^3^) = 0.5 × length × width^2^. At the end of experiment, the tumors, main organs, and blood samples were harvested for analyzing PLK1 mRNA expression, pathological change, and serum biochemistry, respectively. Eight serum biochemistry parameters including triglycerides (TG), total cholesterol (TC), blood urea nitrogen (UREA), total protein (TP), alkaline phosphatase (ALP), alanine aminotransferase (ALT), and aspartate transaminase (AST) were examined.

### 4.15 Anticancer effects of iLNP formulations in patient‐derived xenograft (PDX) model

To establish patient‐derived xenograft (PDX) tumor model, tumor tissue from a liver cancer patient was washed three times with PBS and divided into small pieces, then transplanted into the right armpit of the mice. All procedures were in accordance to principles of the medical ethics and laboratory animal ethics. When the tumors grew to 100–200 mm^3^, mice were randomly divided into six groups and treated with (1) PBS, (2) Sorafenib, (3) GAP35/siPLK1 (i.v.), (4) GARP35/siPLK1 (i.v.), (5) GAP35/siPLK1 (i.t.), and (6) GARP35/siPLK1 (i.t.), respectively. GAP35/siPLK1 was administered intravenously and intratumorally in group 3 and group 5, respectively. GARP35/siPLK1 was also administered intravenously and intratumorally in group 4 and group 6, respectively. siRNA was administered at 1 mg/kg. Sorafenib was dosed orally at 30 mg/kg.

MRI was performed before administration and after the animals received the first dose of testing formulations. Here, the animals were anaesthetized with isoflurane, secured on an animal bed, and placed in a birdcage resonator that was positioned at the correct anatomical location of the animal in a 7.0 T small animal MRI instrument (BioSpec 70/20 USR, Bruker, Germany). T1‐weighted images were acquired before administration and at 1, 5, 10, and 24 h post injection. Meanwhile, body weights, tumor volumes, and animal survival were recorded during the whole treatment course. At the end of experiment, the tumor tissues were isolated, weighted, and optically imaged. The coefficients of the livers or spleens were calculated by dividing the weights of the liver or spleen to the weight of the brain, respectively. The serum biochemistry parameters, as well as the pathological change of the main organs and tumors were also examined.

### 4.16 Statistical analysis

Statistical analysis was performed using GraphPad Prism 8 software. The experimental results were shown as mean ± SEM or mean ± SD. Statistical differences were analyzed with one‐way ANOVA followed by Tukey's test or Student *t*‐test (*P < 0.05, **P < 0.01, ***P < 0.001, ****P < 0.0001).

## CONFLICT OF INTEREST

S.G. and Y.H. declare that a patent relative the study has been filed. The remaining authors declare no competing interests.

## Supporting information

Supporting Information
