# Supplementary material for: Membrane‐destabilizing ionizable lipid empowered imaging‐guided siRNA delivery and cancer treatment
Source: Exploration (Beijing). 2021 Sep 1;1(1):35–49. doi: 10.1002/EXP.20210008 (PMC10291568; doi:10.1002/EXP.20210008)
Supplement: Supplementary file 1 — Supporting Information [file EXP2-1-35-s001.docx]

Supporting Information

**Membrane-destabilizing ionizable lipid empowered imaging-guided siRNA delivery and cancer treatment**

Shuai Guo, Kun Li, Bo Hu, Chunhui Li, Mengjie Zhang, Abid Hussain, Xiaoxia Wang, Qiang Cheng, Feng Yang, Kun Ge, Jinchao Zhang, Jin Chang, Xing-Jie Liang, Yuhua Weng, Yuanyu Huang*

S.Guo, K. Li, B. Hu, C. Li, M. Zhang, Dr. A. Hussain, Prof. Y. Weng, Prof. Y. Huang

School of Life Science; Advanced Research Institute of Multidisciplinary Science; Key Laboratory of Molecular Medicine and Biotherapy; Institute of Engineering Medicine; Beijing Institute of Technology, Beijing, 100081, P. R. China. E-mail: yyhuang@bit.edu.cn

Dr. X. Wang

Institute of Molecular Medicine, College of Future Technology, Peking University, Beijing 100871, China

Dr. Q. Cheng

The University of Texas Southwestern Medical Center, Department of Biochemistry, Simmons Comprehensive Cancer Center, Dallas, TX, USA.

Dr. F. Yang

Howard Hughes Medical Institute, Department of Medicine, School of Medicine, University of California, San Diego, La Jolla, CA 92093, USA.

Prof. K. Ge, Prof. J. Zhang

Key Laboratory of Analytical Science and Technology of Hebei Province, College of Chemistry and Environmental Science, Key Laboratory of Medicinal Chemistry and Molecular Diagnosis of the Ministry of Education, Hebei University, Baoding, 071002, China

Prof. J. Chang,

School of Life Sciences, Tianjin University, Tianjin Engineering Center of Micro Nano Biomaterials and Detection Treatment Technology, Collaborative Innovation Center of Chemical Science and Engineering, Tianjin, 300072, China

Prof. X. Liang

Chinese Academy of Sciences (CAS) Center for Excellence in Nanoscience and CAS Key Laboratory for Biomedical Effects of Nanomaterials and Nanosafety, National Center for Nanoscience and Technology, Beijing, 100190, China


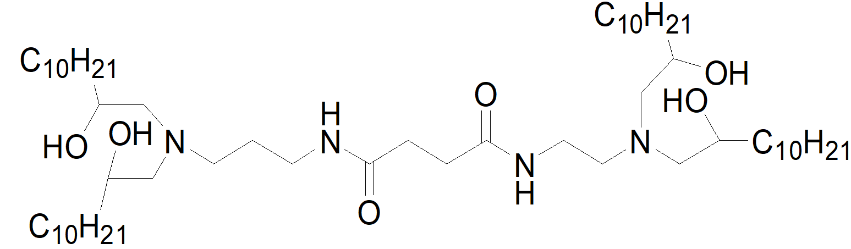


Figure S1. Chemical structure of ionizable lipid iBL0104


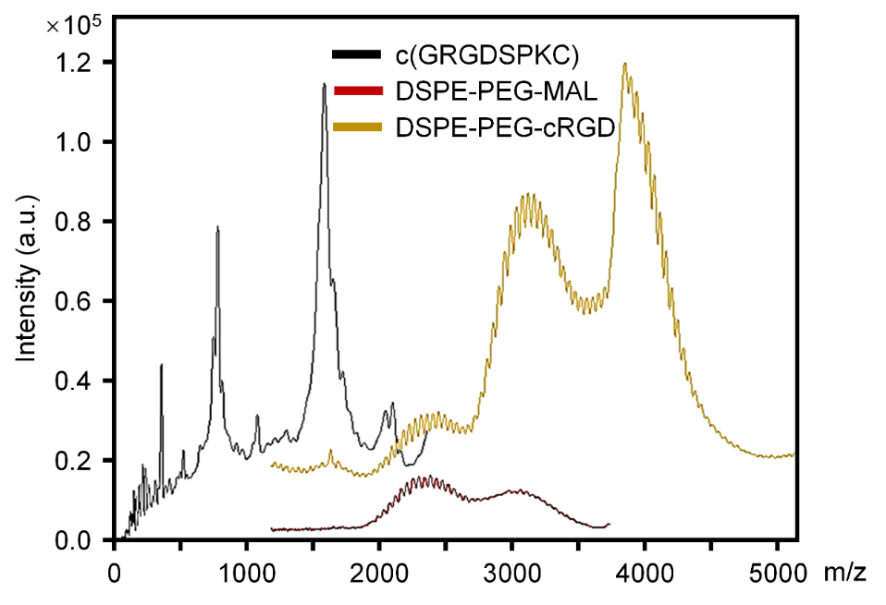


Figure S2. MALDI–TOF–MS analysis of conjugation of cRGD peptide, DSPE-PEG2000-MAL and DSPE-PEG_2000_-cRGD.


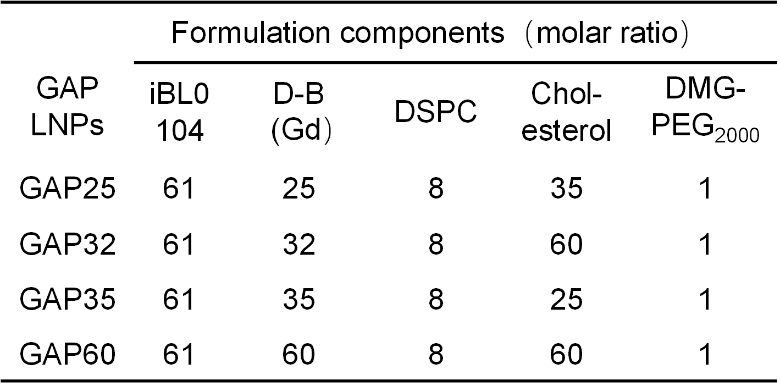


Figure S3. Formulation molar ratios of GAP iLNPs.


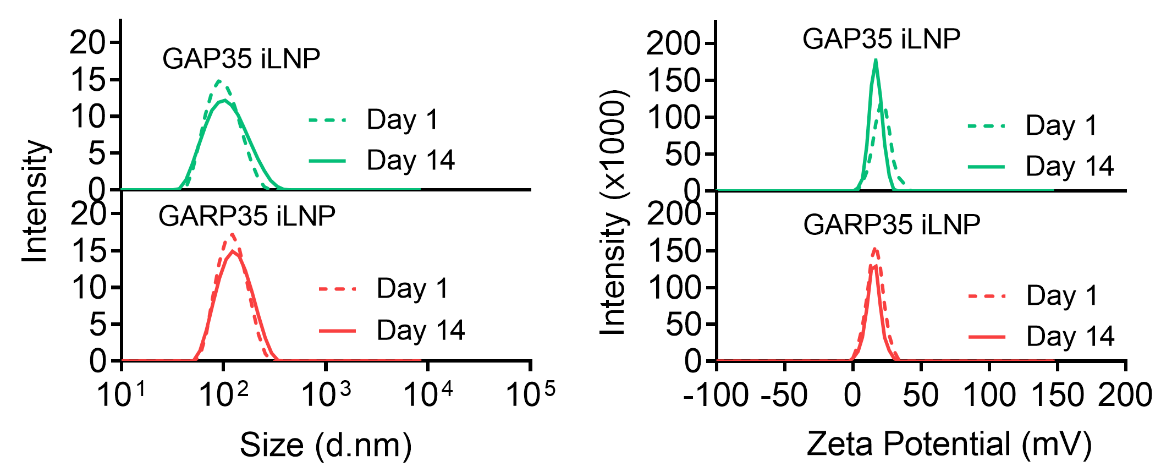


Figure S4. Particle sizes (a) and zeta potentials (b) of GAP35 and GARP35 iLNPs within two weeks.

Figure S5. siRNA encapsulation efficiencies of various iLNPs as detected by RiboGreen assay.


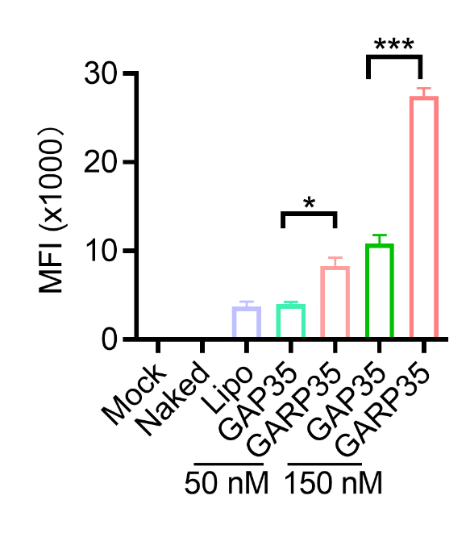


Figure S6. Quantitative analysis of the mean fluorescence intensities of Cy5-siRNA of Figure 2e.


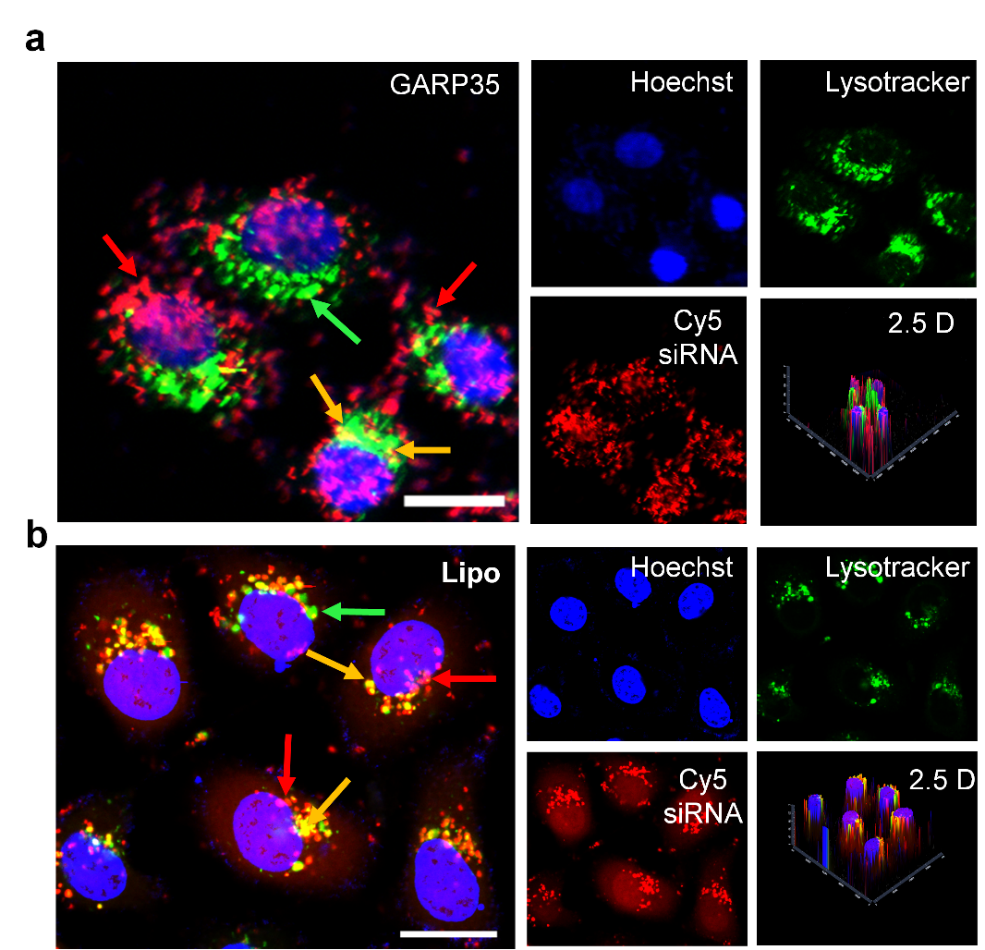


Figure S7. Confocal imaging of HepG2-luc cells transfected with GARP35/Cy5-siRNA (a) and Lipo2000/Cy5-siRNA (b) for 4 h at the final siRNA concentration of 50 nM. Green arrow, endosome/lysosome; red arrow, Cy5-siRNA; yellow arrow, co-localization of endosome/lysosome and Cy5-siRNA. Scale bar, 20 μm.


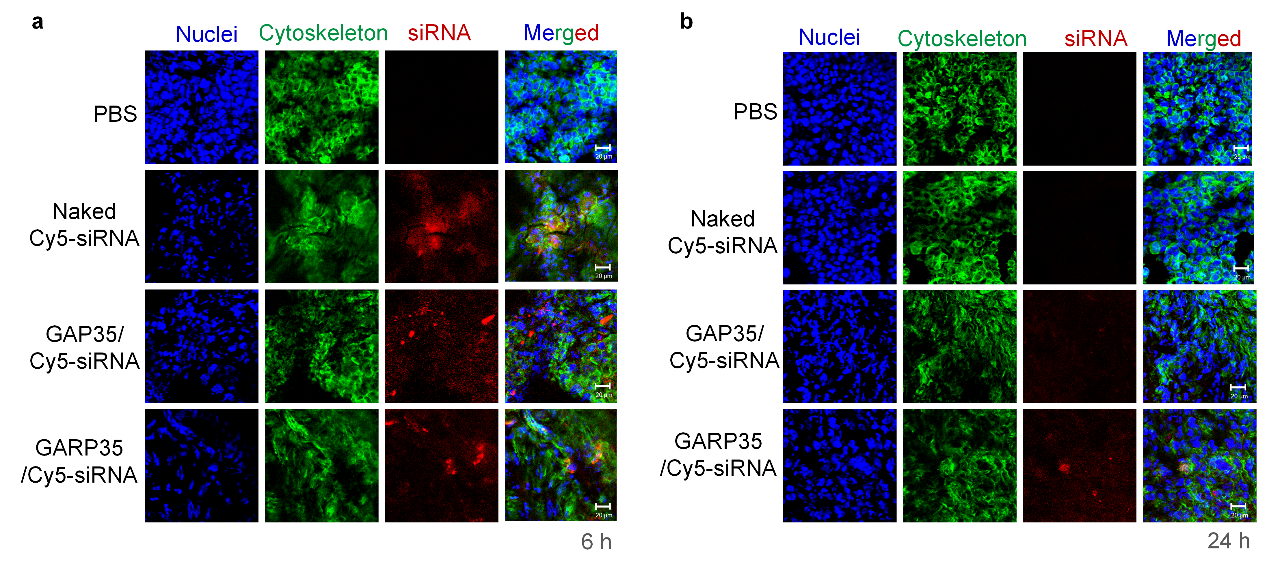


Figure S8. Confocal laser scanning microscopic observation of the cryosections of tumor tissues. (a, b) Tumor section observed 6 h (a) and 24 h (b) after intravenous injection. Scale bar: 20 μm. DAPI and FITC-labeled phalloidin were used to stain the nuclei and F actin, respectively.


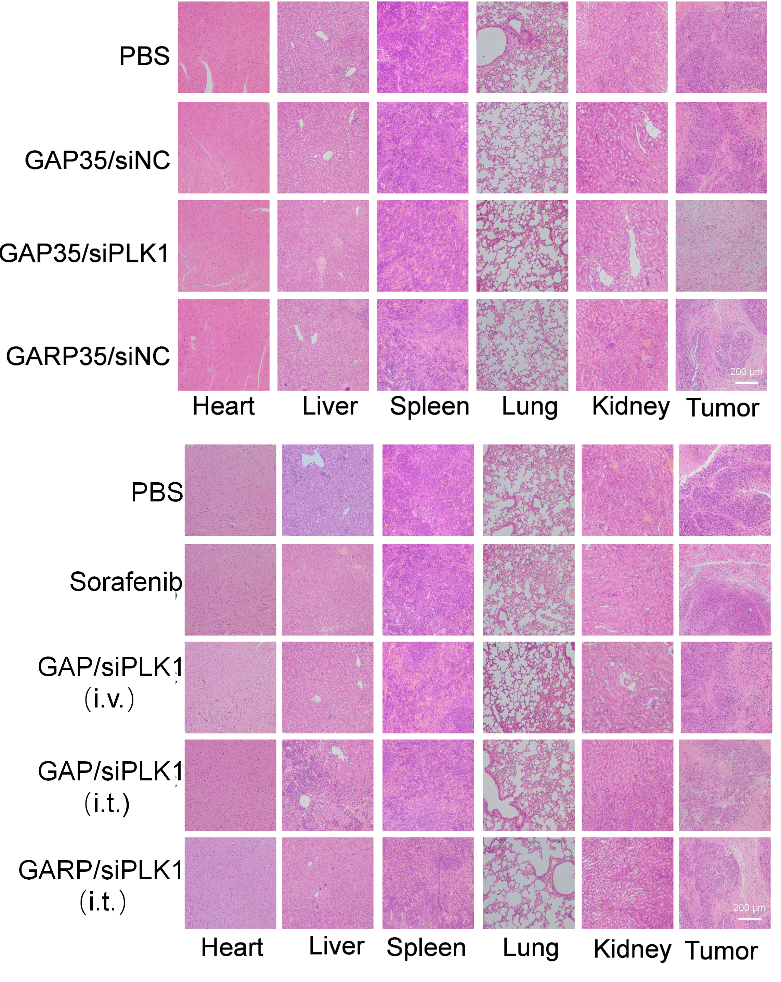


Figure S9. H&E staining of tissue sections prepared with the major organs and tumors collected from the CDX model (top) and PDX model (bottom). Scale bar, 200 μm.
